# The Relationship Between the Managerial Skills and Results of “Performance Evaluation “Tool Among Nursing Managers in Teaching Hospitals of Iran University of Medical Science

**DOI:** 10.5539/gjhs.v7n2p38

**Published:** 2014-09-25

**Authors:** Haleh Mousavi Isfahani, Aidin Aryankhesal, Hamid Haghani

**Affiliations:** 1 School of Public Health, Tehran University of Medical Sciences, Tehran, Iran; 2 Health Management and Economics Research Center, Iran University of Medical Sciences, Tehran, Iran; 3 Faculty of Nursing and Midwifery, Tehran University of Medical Sciences, Tehran, Iran

**Keywords:** performance evaluation, managerial skills, hospital, nursing managers

## Abstract

Performance of different organizations, such as hospitals is mainly influenced by their managers’ performance. Nursing managers have an important role in hospital performance and their managerial skills can improve the quality of the services. Hence, the present study was conducted in order to assess the relationship between the managerial skills and the results of their performance evaluation in Teaching Hospitals of Iran University of Medical Science in 2013. The research used the cross sectional method in 2013. It was done by distributing a managerial skills assessment questionnaire, with close-ended questions in 5 choice Likert scale, among 181 managers and head nurses of hospitals of Iran university of Medical Sciences; among which 131 answered the questions. Another data collection tools was a forms to record evaluation marks from the personnel records. We used Pearson and Spearman correlation tests and SPSS for analysis and description (frequency, mean and standard deviation). Results showed that the managerial skills of the nursing mangers were fair (2.57 out of 5) and the results of the performance evaluation were in a good condition (98.44). The mangers’ evaluation results and the managerial skills scores were not in a meaningful correlation (r=0.047 np=0.856). The research showed no correlation between different domains of managerial skills and the performance evaluation marks: decision making skills (r=0.074 and p=0.399), leadership (correlation coefficient 0.028 and p=0.654), motivation (correlation coefficient 0.118 and p=0.163), communication (correlation coefficient 0.116 and p=0.122), systematic thinking (correlation coefficient 0.028 and p=0.828), time management (correlation coefficient 0.077 and p=0.401) and strategic thinking (correlation coefficient 0.041 and p=0.756). Lack of any correlation and relation between managers’ managerial skills and their performance evaluation results shows need to a fundamental revision at managers’ performance evaluation form.

## 1. Introduction

Nowadays human resources are of the most valuable factors of the production and in all organizations count as the most important capital and producer of the main qualifications. In health sector also manpower is the fundamental capital of any organization (Mirzasadeghi, 2001). Managers, in current complicated organizations, lay the most important role among the human workforce and have the most effective role in improving organizations’ performance (Gerber, Nel, & Van Dyk, 1995). On the other hand, the efficiency of managers is associated with managerial skills that enable them for different roles and responsibilities. Thus, beside the knowledge and experience, managers should gain some special skills (Bazargan Harandi, 2003). Such skills are at greater importance when dealing with health organizations, especially hospitals, because they are responsible of peoples’ lives (Akbari, Tofighi, Torabi, Arab, & Tarahi, 2005). Also, in hospitals nursing managers, in comparison to other stuffs, have more critical role in the progress and performance of the organization and developing the quality of the services (Vafaee Najar, Habashizade, Karimi, & Ebrahimzade, 2011). Researches show that the nursing managers lack the effective managerial skills (Heath, Johanson, & Blake, 2004). It is the performance evaluation that makes these skills better and prepares the ground ready to develop them. Performance evaluation analyses the managers’ skills and performance by determining their strenghts and weaknesses. In fact, performance evaluation counts as a tool for developing managerial skills (Asgharizade, Ehsani, & Valipour Halabi, 2012; Pazargadi, Afzali, Javadzadeh, & Alavi Majd, 2005).

Performance evaluation is a process in which the performance of personnel is assessed and analyzed formally in given intervals and is one of the qualifications of the present professional world which can be helpful in hiring qualified staff (Pazargadi, Ashktorab, Alavimajd, & Khosravi, 2013; Najafi, 2010). Also, performance evaluation is one of the most practical and best methods for solving problems and developing managers’ skills (Azamy, 2000). In our country (Iran) all staff get evaluated for their performance annually, and the nursing managers are not the exception, either. However, the present evaluations in the healthcare systems of our country are not organized and precise (Pazargadi et al., 2013).

Studies have shown that the results gained in our society are in medium level practically and efficiently and their reliability and importance is very poor (Gerber, Nel, & VanDyk, 1995). In fact there is no clear and comprehensive process for evaluating the performance of the medical society in order to resolve the problems, create motivation and suitable substructure in order to improve the performance (Sheikhzadeh, 2010).

Owing to the weakness and defects of the performance evaluation systems in our hospitals (Khatiban, Pazargadi, & Ashktorab, 2011; Pazargadi et al., 2005; Sheikhzadeh, 2010; Janati, 2012; Yarmohammadian, Bahrami, Karimian, Shahrzadi, & Mosadegh rad, 2005), we do not expect to get meaningful correlation between nursing managers’ managerial skills and their annual score resulted from performance evaluation tool. Nevertheless no study has examined such relationship so far. So this study by examining such relationship would try to answer if nursing managers’ performance resulted from evaluation tools is related with their managerial skills.

## 2. Method

This descriptive analytical study was done cross sectionally in 2013. The research sample included all matrons and headnurses of teaching hospitals of Iran University of Medical Sciences. The research units were selected by census of all 181 people who agreed to attend the study. The research tools included a form for collecting the evaluation marks of nursing managers and a questionnaire for examining respondents’ managerial skills. For getting the performance evaluation marks of nursing managers in 2012, researchers extracted the necessary information from the nursing managers’ personnel files. For evaluating the managerial skills they distributed a close questionnaire with some statements about managers’ actions in certain occasions followed by a 5 – choice Likert scale answer including completely disagree, disagree, no idea, agree and completely agree. The professionals of the healthcare managers confirmed the conceptual and exterior validity of the questionnaire. For reviewing the reliability of the questionnaire, first we distributed them among 30 people and then asked them to answer the questionnaire again two weeks later. Conducting a correlation test of Cronbach, we got a correlation of 74%.

The questionnaire included two sections, one for getting personal information and the other for the main part which included 24 questions about managerial skills in 7 subsequent (decision making, communication, leadership, motivation, systematic thinking, time management and strategic thinking skills). The researcher distributed the questionnaire among the nursing managers at hospitals of Iran University of Medical Sciences. Among these hospitals two hospitals were deleted from the list, because of unwillingness to attend the survey. Also since all supervisors were busy and just a few of them agreed to attend the study, we omitted them from the sample society. Finally 131 questionnaires were collected from the 181. For analyzing the collected data and confirming the hypothesis, we used the descriptive and analytical statistical methods. Data were analyzed in SPSS. For the descriptive statistics we had the tables of the frequency distribution, percent, mean and standard deviation. Since the statistical sample society was abnormal, for determining the correlation between the variables we used the nonparametric statistics (Spearman correlation coefficient). This correlation coefficient was applied to find the correlation between the people’s rank in evaluation marks and their skill grades.

## 3. Results

The characteristics of study respondents are summarized at Table 1. Among the 131 participants in this study, 85.6 % were women and the others were men. More than half of them were between 40 to 49 years old (53.4) and the least age group was for the people less than 30 (3.1); according to these results the average age of respondents was 41. 23 (SD=0.68). Most of the managers and the headnurses attending the survey had a BS degree (83.9%). The least frequency for the work experience was less than 5 years (21.4%) and its most amounts were between 15 to 25 years (35.1%), so the average working experience was 17.23 years with a standard deviation of 6.93.

**Table 1:** Demographic characteristics of the managers and headnurses in the given studied hospitals

Demographic variables		frequency	Frequency distribution
age	Less than 30 years	4	3.1
	Between 30 to 40 years	45	34.4
	Between 40 to 49 years	70	53.4
	More than 50 years	12	9.2

gender	Female	112	85.6
	Male	19	14.2

education	Basic degree	1	0.08
	B.A	110	83.9
	M.A	20	15.3

Working experience	Less than 5 years	28	21.4
	5 to 15 years	35	26.7
	Between 15 to 25 years	46	35.1
	More than 25 years	22	16.8

Table 2 shows the respondents managerial skills scores, resulted in from their answers to the questionnaire. As shown in Table 2, “decision making skill” with a mean of 2.8 (out of 5) had the best mean and the “motivation skill” with 2.55 (of 5) had the least mean.

**Table 2:** Mean and the standard deviation of managerial skills and their dimensions

Variable	Mean (out of 5)	Standard Deviation
Decision making skill	2.8	0.47
Communication skill	2.76	0.67
Time management skill	2.75	0.56
Strategic thinking skill	2.73	0.56
Systematic thinking skill	2.66	0.61
Leadership skill	2.56	0.57
Motivation skill	2.55	0.62
All the managerial skills	2.56	0.23

As shown in Table 3, the total mean of the performance evaluation marks among 131 participants was 89.44 (out of 100) and the standard deviation of this variable was 3.93. HospitalE with the mean of 86.3 and the standard deviation of 3.95 had the least mark and hospital D with the mean of 93.3 and the standard deviation of 2.97 got the best mark.

**Table 3:** Mean and the standard deviation of performance evaluation of the managers in each hospital

Name of the hospital	Average of the Performance evaluation mark (out of 100)	Standard deviation of the performance evaluation mark
Hospital A	88.2	3.89
Hospital B	89.8	3.97
Hospital C	01.4	3.46
Hospital D	93.3	2.97
Hospital E	86.3	3.95
Hospital F	87.3	3.91
Hospital G	92.1	3.42
Hospital H	90.5	3.54
Total Mean	89.44	3.39

As shown in Table 4, there is not a meaningful correlation between the managerial skills and its different domains with the results of the performance evaluation.

**Table 4:** The correlation between the managerial skills marks and the performance evaluation

Variable	Performance evaluation	

	Spearman Correlation	P-value
Decision making skill	0.74	0.399
Leadership skill	0.028	0.654
Motivation skill	0.118	0.163
Communication skill	0.116	0.122
Systematic thinking skill	0.028	0.828
Time management skill	0.077	0.401
Strategic thinking skill	0.041	0.756
All the managerial skills	0.47	0.856

## 4. Discussion

Total mean of the managerial skill marks among 131 nursing mangers and headnurses was 2.56 (of 5) and the mean for the performance evaluation of the hospitals was 89.44 (of 100), with no meaningful correlation between the two varriable.

Results of the study convey that the level of the managerial skills among respondents was less than the average. In the study of Mohsenpour (2011) the managerial level of the managers was low; these results in both of these studies follow the same conclusion. Mohsenpour points that managerial skills of the managers are an important factor for improving the work proficiencyand lack of these skills causes disorders in the organization. This study also emphasizes on the importance of developing the managerial skills of the nursing managers. Zaimipur (2006) also did a survey about the effect of reinstructing the managerial skills for nursing managers in order to enabling them, and points to the weaknesses of the managerial skills needing reinforcement. These entire conclusions are in harmony with our findings. He concluded that the reinstruction program of managerial and leadership skills for the nursing managers enables the organizational and psychological abilities of the personnel and improves their professional performance. Kerridge, Supic and Lin in their study also emphasized on improving the managerial skills of the healthcare centers, the same as the present study (Kerridge, 2012; Lin, Wu, Huang, Tseng, & Lawler, 2007; Supic, Bjegovic, Marinkovic, Milicevic, & Vasic, 2010).

The results of the present study didn’t show any meaningful correlation between the managerial skills of the nursing managers and their performance evaluation marks.

Hamidi in 2011 studied the relationship between the managerial skills and the job stress and conveyed that there is a negative correlation between the job stress and the humanistic, perceptual and technical skills of the managers. In other words the study showed that the level of professional skills of the managers had a strong relationship with the stress of the staff. This study stated that teaching and developing the managerial skills of the supervisors and top managers in healthcare centers plays an important role in decreasing the job stress among staff and improving the performance and efficiency of the organization (Hamidi, Mehri, Zamanparvar, & Imani, 2012). As the study emphasizes on the importance of instructing the managerial skills in healthcare departments, the weakness in this field is confirmed. These findings also are the same as our findings in the present study. Zaimipur in 2004 conducted a research about the influence of the reinstructing the managerial competencies of the nursing managers on enabling the nursing personnel and concluded that the program of reinstructing the managerial skills improves and develops the personnel professional performance. This strong effect of teaching the managerial skills emphasizes on the weakness of the mangers’ managerial skills and matches with our findings in this study.

Findings of the present study showed that the mean of the performance evaluation of the nursing managers was 89.44 which is a good score, but lack of correlation between this variable and the managerial skills could be a sign of weakness in the evaluation system of the managers’ performance. Zaboli conducted a study under the title of assessing the performance evaluation system of the managers in hospitals of the Iran University of Medical Sciences and found the same results. According to the results of Zaboli’s study, the system of the performance evaluation of the staff did not match the official system of the hospitals and the social and cultural realities which causes dissatisfaction among the personnel from the evaluation system (Zaboli, Delgoshaei, & Haghani, 2005). According to the findings of Hamidi and Khatibiyan (Khatiban et al., 2011; Hamidi, 2009), the performance evaluation system was not effective and reforming its content and performance was suggested. Pazargadi also states that there is a negative view about the present performance evaluation between the managers and head nurses (Pazargadi et al., 2005). Nikpeyma in 2013 also concluded that there was lack of objectivity in the present performance evaluation system; he stated that this system had general and unskilled principles not matching the realities of the administrative system. Since the study points to the generality of the performance evaluation system, it confirms the weakness of the system. Among the foreign studies we can point to the Redshaw’s; according to which performance evaluation system is in fact some forms just filled after the interviews and are not revised or get feedback later. In fact this system of evaluation is a kind of bureaucratic process that creates negative views among staff (Redshaw & Man, 2008).

As limitations of our study, in some of the hospitals it was difficult to arrange the time, because of the long administrative process; and because of problems we faced in data collection. On the other hand, since the study was not practical for improving the work conditions, some of the managers did not tend to answer the questions; and if answered, their answers were not filled carefully. Also the respondents did not trust us to consider the privacy of their information. Although in this study we ensured the respondents about the privacy of the information, some may have answered questions dishonestly. We should consider that dishonesty in answering the questions is one of the gaps in most researches.

## 5. Conclusion

Although the relationship between managerial skills and the results of the performance evaluation should be a logical expectation, this study does not confirm such logical relationship. Our findings show that there is no correlation between the managerial skills of the nursing managers and the results of the performance evaluation in different skills’ domains. When the managerial skills of the managers were less than the average, but the mean for the performance evaluation of the managers was good we can conclude that there is a weakness in the performance evaluation system, regarding that our managerial skills measurement tool was a valid one. Of the most practical and best ways to solve the problems and improve the managerial skills and competencies is performance evaluation (Azamy, 2000). But findings of the research, like all the other researches in past 10 years, confirm that the performance evaluation system has no influence among the medical society. Despite many researches done in the recent years about the system of managers’ performance evaluation which conveys its effect, no fundamental changes have been done and the results of the surveys one after another shows the problems in solving the problems of the system of performance evaluation. Necessity of fundamental changes and revision in the system of managers’ performance evaluation and its integration with the scientific principles of management is essential. Also since managers of the organizations have the most important role in improving the organization’s performance, considering the system of performance evaluation and determining the factors, indexes and new methods of performance evaluation plays an important role. Holding educational courses can help reinforcing the managerial skills of the nursing managers. We may consider that paying attention and focusing of the undertakers on the nature of the research and its results can solve many of the problems.
